# Comparative Performance of ^68^ Ga-FAPI-04 and ^18^ F-FDG PET/CT in Ovarian Cancer: Focus on Peritoneal Carcinomatosis

**DOI:** 10.1055/s-0046-1819631

**Published:** 2026-03-20

**Authors:** Esra Arslan, Göksel Alçin, Nurhan Ergül, Elife Akgün, Ömer Faruk Şahin, Zehranur Tosunoğlu, Ahmet Ertuğrul Öztürk, Tevfik Fikret Çermik

**Affiliations:** 1 Department of Nuclear Medicine, University of Health Sciences, Istanbul Training and Research Hospital, Istanbul, Türkiye; 2 Department of Nuclear Medicine, Fakeeh University Hospital, Dubai, United Arab Emirates; 3 Department of Nuclear Medicine, University of Health Sciences, Ümraniye Training and Research Hospital, Istanbul, Türkiye; 4 Department of Nuclear Medicine, Acıbadem Maslak Hospital, Istanbul, Türkiye

**Keywords:** ^68^ Ga-FAPI-04 PET/CT, ^18^ F-FDG PET/CT, ovarian cancer, peritoneal carcinomatosis, cancer-associated fibroblasts, fibroblast activation protein inhibitor, fibroblast activation protein

## Abstract

**Objective:**

To evaluate the diagnostic performance of ^68^ Ga-FAPI-04 positron emission tomography/computed tomography (PET/CT) in ovarian cancer compared with ^18^ F-FDG PET/CT, focusing on peritoneal carcinomatosis.

**Materials and Methods:**

Twelve patients with histologically confirmed ovarian cancer underwent both ^68^ Ga-FAPI-04 and ^18^ F-FDG PET/CT within 1 week for staging, restaging, or recurrence assessment. SUVmax values of primary tumors, lymph nodes, and peritoneal metastases were compared. Tumor-to-background ratios were calculated, and statistical analyses were performed using nonparametric tests with *p* <0.05 considered significant.

**Results:**

High-grade serous carcinoma was the predominant histology (83.3%). Lymph node metastases were detected in 7/12 patients (58.3%), including distant nodes in 3/12 (25.0%), locoregional nodes in 2/12 (16.7%), and combined disease in 2/12 (16.7%). Peritoneal carcinomatosis was observed in 9/12 patients (75.0%), with significantly higher SUVmax on ^68^ Ga-FAPI-04 compared with ^18^ F-FDG (19.17 ± 9.13 vs. 12.41 ± 7.13, *p* = 0.016). No significant differences were observed in nodal metastases or distant sites. In 4 of 12 patients (33.3%), ^68^ Ga-FAPI-04 PET/CT identified additional lesions not visualized on ^18^ F-FDG PET/CT.

**Conclusion:**

^68^ Ga-FAPI-04 PET/CT demonstrated superior peritoneal carcinomatosis detection compared with ^18^ F-FDG PET/CT in ovarian cancer. Although derived from a small single-center cohort, these findings are consistent with emerging evidence and highlight the potential role of FAPI imaging in improving staging and management.

## Introduction

Epithelial ovarian cancer remains the most lethal gynecological malignancy, primarily due to its late presentation and frequent peritoneal dissemination at diagnosis. 1 Accurate assessment of peritoneal carcinomatosis (PC) is crucial for staging, determining resectability, and guiding optimal treatment strategies. Conventional imaging modalities, including contrast-enhanced computed tomography (CT) and magnetic resonance imaging, are recommended in consensus guidelines but have limited sensitivity in detecting small-volume peritoneal disease. 2 3 ^18^ F-FDG positron emission tomography (PET)/CT, while widely used in oncologic imaging and supported by multidisciplinary recommendations, 4 5 also shows restricted performance in ovarian cancer because of variable tumor glycolytic activity, physiological uptake in ovaries related to the menstrual cycle, and limited contrast in diffuse peritoneal spread. 6

Fibroblast activation protein (FAP) is highly expressed in cancer-associated fibroblasts, which constitute a major component of the tumor stroma. Radiolabeled fibroblast activation protein inhibitors (FAPIs) have emerged as promising PET tracers with rapid tumor uptake and low background activity. The pioneering clinical study by Kratochwil et al 7 established the high tumor-to-background contrast of FAPI PET across multiple tumor types, including ovarian cancer. Translational data have confirmed that tracer uptake correlates with stromal FAP expression, 8 providing a robust biological rationale for clinical application. Early ovarian-focused studies further demonstrated that FAPI PET/CT detects PC more sensitively than FDG and yields higher Peritoneal Cancer Index (PCI) scores. 9 10 11

More recently, medium and large-scale studies have consistently shown the superiority of FAPI PET/CT in primary staging and restaging ovarian cancer, with direct implications for surgical planning and treatment decision-making. 12 13 14 15 16 High-level syntheses, including two meta-analyses 17 18 and an integrative review, 19 have confirmed these findings, consistently reporting superior FAPI PET/CT sensitivity over FDG for peritoneal and nodal metastases in ovarian cancer.

Given this background, we conducted a prospective study in 12 patients with ovarian cancer to compare the diagnostic performance of ^68^ Ga-FAPI-04 PET/CT with ^18^ F-FDG PET/CT, focusing on the evaluation of primary tumors, lymph node (LN) involvement, and peritoneal metastases. The aim was to investigate whether FAPI imaging provides added diagnostic value and could serve as a clinically relevant adjunct to FDG in the staging and follow-up of ovarian malignancies.

## Materials and Methods

### Patients

Twelve patients with histologically confirmed ovarian cancer who underwent both whole-body ^68^ Ga-FAPI-04 PET/CT and ^18^ F-FDG PET/CT within the same week for staging, restaging, or recurrence assessment were prospectively enrolled. The mean age was 58.3 ± 11.9 years (range: 41–76 years). The study was approved by the local institutional review board, and all procedures were conducted in accordance with the ethical standards of the Declaration of Helsinki. Written informed consent was obtained from all patients for the use of their clinical data for research purposes.

### Histopathology

Histopathological evaluation was performed by an experienced pathologist blinded to PET/CT results. Samples obtained from the uterus, ovaries, omentum, peritoneal biopsies, and pelvic-aortic LNs were stained with hematoxylin–eosin and reviewed microscopically. Recurrence was diagnosed either by histopathology (surgery, laparotomy, or biopsy) or clinical follow-up for at least 6 months, including FDG PET/CT performed in cases with sustained elevation of CA-125 to twice the upper limit of normal.

### ^18^ F-FDG PET/CT

Patients fasted for at least 6 hours before tracer administration; only those with blood glucose <150 mg/dL were included. A standard dose of 3.7 to 5.2 MBq/kg 18F-FDG was administered intravenously. Whole-body PET/CT scans (mCT 20 ultraHD LSO PET/CT; Siemens Molecular Imaging, Hoffman Estates, Illinois, United States) were acquired from the vertex to the mid-thigh 60 minutes post-injection. SUVmax was measured using a volume of interest (VOI) placed over the area of highest tracer uptake.

### ^68^ Ga-FAPI-04 PET/CT

Patients received an intravenous injection of 185 MBq (range: 52–325 MBq) of ^68^ Ga-FAPI-04. Whole-body PET/CT imaging was performed under identical acquisition parameters to FDG, beginning 60 minutes post-injection. SUVmax was measured using VOIs drawn over the most avid lesion sites. Tumor-to-background ratios (TBRs) were calculated by normalizing lesion SUVmax to the ascending aorta blood pool SUV.

### Statistical Analysis

All analyses were performed using SPSS (v21.0; IBM, Armonk, New York, United States). Continuous variables were expressed as mean ± standard deviation, median (range), or percentages, as appropriate. Paired comparisons between ^68^ Ga-FAPI-04 and ^18^ F-FDG measurements were conducted using the Wilcoxon signed-rank test, as variables were nonnormally distributed. A *p* -value <0.05 was considered statistically significant.

## Results

Twelve patients with ovarian cancer (mean age 58.3 ± 11.9 years; range 41–76) underwent both ^68^ Ga-FAPI-04 and ^18^ F-FDG PET/CT. Imaging indications were staging in seven patients (58.3%) and restaging in five patients (41.7%). Histology was high-grade serous carcinoma in 10/12 (83.3%), with 1 case each of low-grade papillary serous and clear cell carcinoma.

**Fig. 1:**
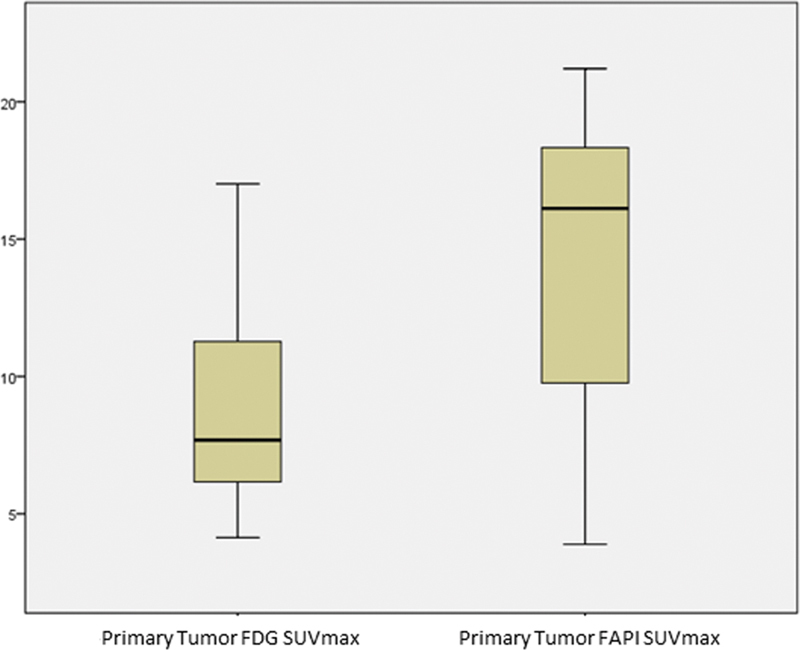
Boxplot comparison of SUVmax values between ^18^ F-FDG and ^68^ Ga-FAPI-04 in primary ovarian tumors. ^68^ Ga-FAPI-04 PET/CT demonstrates higher median SUVmax than ^18^ F-FDG PET/CT ( *p* = 0.028). CT, computed tomography; FAPI, fibroblast activation protein inhibitor; PET, positron emission tomography.

Quantitatively, ^68^ Ga-FAPI-04 demonstrated higher SUVmax values than ^18^ F-FDG in primary ovarian tumors ( Fig. 1 ). No significant between-tracer differences were observed for locoregional nodal metastases ( *p* = 0.285), distant nodal metastases ( *p* = 0.144), and bone metastasis ( Table 1 ).

**Table 1:** Comparison of SUVmax values between ^18^ F-FDG and ^68^ Ga-FAPI-04 ( *N* = 12)

Lesion site	^18^ F-FDG SUVmax (mean ± SD)	^68^ Ga-FAPI-04 SUVmax (mean ± SD)	*p* -Value
Primary tumor	8.94 ± 4.30	13.68 ± 6.17	0.028 a
Locoregional lymph nodes	11.91 ± 4.30	10.53 ± 4.62	0.285
Distant lymph nodes	4.80 ± 2.60	9.13 ± 2.32	0.144
Bone	8.64	34.50	–

Abbreviations: FAPI, fibroblast activation protein inhibitor; SD, standard deviation.

a *p* <0.05 statistically significant. Statistical comparisons were performed using the Wilcoxon signed-rank test.

**Fig. 2:**
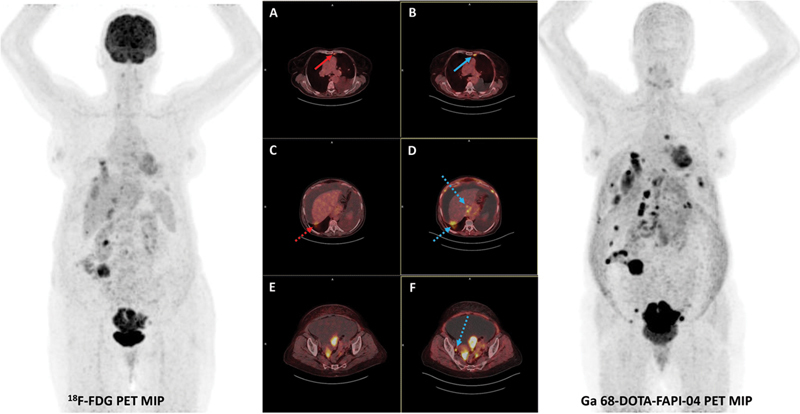
A 71-year-old woman with a metastatic lymph node located in the left parasternal region (FAPI SUVmax = 10.3, FDG SUVmax = 3.9). ( **A** ) The red arrow indicates the lesion on the fused ^18^ F-FDG PET/CT image. ( **B** ) The blue arrow indicates the same lesion on the fused ^68^ Ga-FAPI-04 PET/CT image. A low level of uptake was observed with ^18^ F-FDG PET/CT in the posterior right lobe capsule of the liver, while more intense FAPI uptake was noted; ( **C** ) the red dashed arrow on ^18^ F-FDG PET/CT and ( **D** ) blue dashed arrows on ^68^ Ga-FAPI-04 PET/CT. Additionally, a right internal iliac 0.7 cm metastatic LN showed FAPI positivity (SUVmax = 7.4) but was FDG-negative, ( **E, F** ) blue dashed arrows on ^68^ Ga-FAPI-04 PET/CT. CT, computed tomography; FAPI, fibroblast activation protein inhibitor; LN, lymph node; PET, positron emission tomography.

**Fig. 3:**
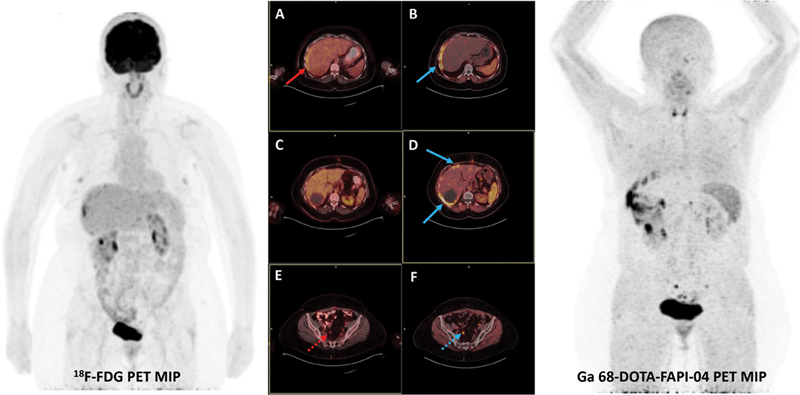
A 48-year-old woman in whom FAPI imaging demonstrated superior visualization of PC (FAPI SUVmax = 30.3, FDG SUVmax = 12.7). ( **A** ) The red arrow indicates the lesion on the fused ^18^ F-FDG PET/CT image; ( **B–D** ) blue arrows indicate corresponding lesions on ^68^ Ga-FAPI-04 PET/CT. FAPI uptake was also more intense than FDG in pelvic metastatic LNs (FAPI SUVmax = 16.7, FDG SUVmax = 5.5); ( **E** ) red dashed arrow on ^18^ F-FDG PET/CT; ( **F** ) blue dashed arrow on ^68^ Ga-FAPI-04 PET/CT. CT, computed tomography; FAPI, fibroblast activation protein inhibitor; LN, lymph node; PC, peritoneal carcinomatosis; PET, positron emission tomography.

**Fig. 4:**
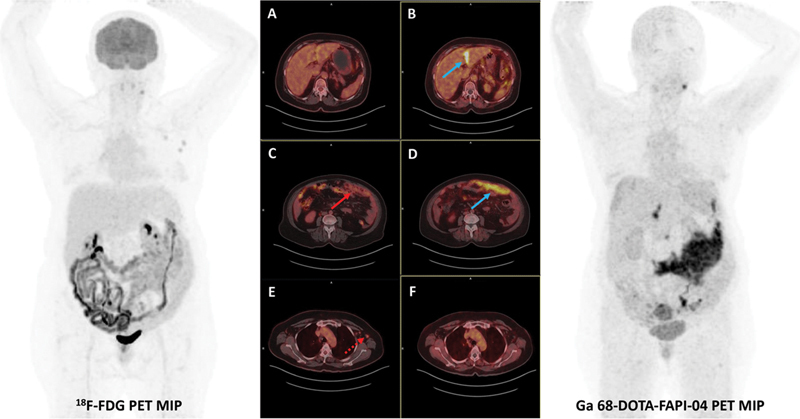
A 57-year-old woman showing low FDG uptake (SUVmax = 3.9) on the liver capsule surface but intense uptake with FAPI (SUVmax = 15.1), ( **A, B** ) blue arrow on ^68^ Ga-FAPI-04 PET/CT. Markedly increased FAPI uptake was also seen on the intestinal serosal surfaces in the left lower quadrant (FAPI SUVmax = 20.7; Bowel SUVmax = 3.6) compared with FDG (FDG SUVmax = 5.9; Bowel SUVmax = 9.2); ( **C** ) red arrow on ^18^ F-FDG PET/CT and ( **D** ) blue arrow on ^68^ Ga-FAPI-04 PET/CT. In addition, although uptake was detected with FDG in the right axillary lymph node, ( **E, F** ) the red dashed arrow on ^18^ F-FDG PET/CT, no significant uptake was observed with FAPI. Biopsy results were consistent with a reactive hyperplastic lymph node (FAPI SUVmax = 1.1, FDG SUVmax = 4.4). CT, computed tomography; FAPI, fibroblast activation protein inhibitor; PET, positron emission tomography.

LN involvement was identi19-03-2026fied in 7/12 patients (58.3%) by both tracers, comprising distant nodal metastases in 3/12 (25.0%), locoregional nodes in 2/12 (16.7%), and combined locoregional plus distant disease in 2/12 (16.7%) ( Fig. 2 ). PC was detected in 9/12 patients (75.0%) with both modalities ( Figs. 3 and 4 ).

**Fig. 5:**
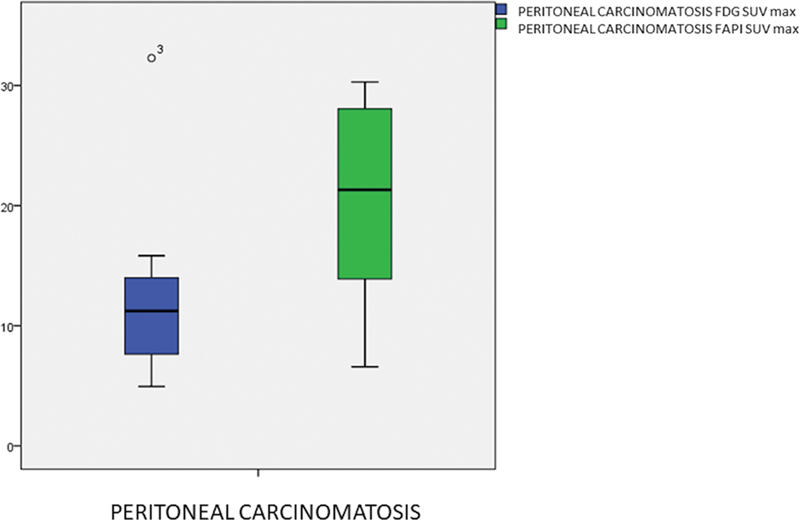
Boxplot comparison of SUVmax values for PC lesions between ^18^ F-FDG and ^68^ Ga-FAPI-04 PET/CT. ^68^ Ga-FAPI-04 shows significantly higher uptake (mean ± SD = 19.17 ± 9.13) compared with ^18^ F-FDG (12.41 ± 7.13; *p* = 0.016). CT, computed tomography; FAPI, fibroblast activation protein inhibitor; PC, peritoneal carcinomatosis; PET, positron emission tomography; SD, standard deviation.

The mean ^68^ Ga-FAPI-04 SUVmax for PC was significantly higher than ^18^ F-FDG (19.17 ± 9.13 vs. 12.41 ± 7.13; *p* = 0.016) ( Fig. 5 ). TBR was numerically higher with ^68^ Ga-FAPI-04 (2.07 vs. 2.02), though not statistically significant ( *p* = 0.060). Blood-pool activity (ascending aorta) was similar between tracers (2.02 ± 0.60 vs. 2.14 ± 0.63; *p* = 0.433) ( Table 2 ).

**Table 2:** Peritoneal carcinomatosis SUVmax, TBR, and blood pool values ( *N* = 9/12 with PC)

Parameter	^18^ F-FDG (mean ± SD)	^68^ Ga-FAPI-04 (mean ± SD)	*p* -Value
Peritoneal carcinomatosis SUVmax	12.41 ± 7.13	19.17 ± 9.13	0.016 a
Tumor-to-background ratio (TBR)	2.02 ± 1.05	2.07 ± 2.67	0.060
Ascending aorta blood pool SUVmax	2.14 ± 0.63	2.02 ± 0.60	0.433

Abbreviations: FAPI, fibroblast activation protein inhibitor; PC, peritoneal carcinomatosis; SD, standard deviation.

a *p* <0.05 statistically significant. Statistical comparisons were performed using the Wilcoxon signed-rank test.

In 4 of 12 patients (33.3%), ^68^ Ga-FAPI-04 PET/CT identified additional lesions that were not visualized on ^18^ F-FDG PET/CT.

Subgroup analyses showed no significant differences in primary-tumor SUVmax according to either tracer's presence or absence of nodal metastases or PC.

## Discussion

In this prospective series of 12 patients with ovarian cancer, we demonstrated that ^68^ Ga-FAPI-04 PET/CT provides superior detection of primary ovarian tumors and PC compared with ^18^ F-FDG PET/CT, with significantly higher SUVmax values in primary lesions and, in patients with PC, markedly higher uptake in peritoneal deposits. These findings highlight the potential of FAPI imaging to overcome the well-recognized limitations of FDG in ovarian malignancies, particularly for assessing diffuse peritoneal spread, which remains a critical determinant of operability and prognosis.

Our results are in line with earlier clinical evidence. The initial landmark work by Kratochwil et al 7 established the uptake profile of FAPI across 28 tumor types and highlighted ovarian cancer as an intermediate-uptake entity with high tumor-to-background contrast due to negligible physiologic pelvic uptake. Subsequent studies confirmed the added diagnostic value of FAPI over FDG in PC: Zhao et al 9 and Dendl et al 10 both reported superior lesion detection and higher PCI scores with FAPI, while Wang et al 11 demonstrated that FAPI avoids the pitfalls of menstrual-cycle-related ovarian uptake that frequently confound FDG interpretation. Translational work further reinforced this biological rationale: Mona et al 8 showed a strong correlation between FAPI uptake and FAP expression by immunohistochemistry, validating FAPI PET as a faithful in vivo biomarker of stromal biology.

More recent comparative studies have consistently confirmed these advantages in ovarian cancer. Zheng et al 20 reported higher detection rates for primary tumors, LNs, and peritoneal or pleural metastases with FAPI, along with upstaging in nearly one-fifth of patients. Liu et al 12 and Chen et al 13 provided prospective evidence in recurrent and newly diagnosed epithelial ovarian cancer, showing that FAPI not only achieved markedly higher sensitivity for PC (∼95–98% vs. 48–76% with FDG) but also altered surgical planning and treatment decisions in a significant proportion of cases. The clinical utility of these findings is underscored by their correlation with surgical PCI and tumor marker levels (CA-125, HE4). Complementary results from Güzel and Kaplan 21 further demonstrated FAPI's ability to delineate peritoneal involvement in ovarian primaries and breast cancer metastatic deposits.

Larger cohorts published more recently have consolidated these observations. Liu et al 16 and Tian et al 15 reported in 79 and 88 patients, respectively, that FAPI significantly improved accuracy for peritoneal, nodal, and recurrent disease compared with FDG, with management modification rates ranging from 10 to 17%. Jiang et al 14 further demonstrated the strong correlation of FAPI-derived PCI with CA-125 and treatment response, while Li et al 22 highlighted its superior specificity in LN assessment and its potential to reduce unnecessary biopsies. Collectively, these findings indicate that FAPI imaging is not only diagnostically superior but also clinically impactful in guiding therapy.

The strength of this evidence has been reflected in systematic syntheses. Two independent meta-analyses, Sun et al 17 and Florit et al, 18 confirmed the consistent superiority of FAPI over FDG in ovarian cancer, particularly for PC (pooled sensitivity ∼97% vs. 70% with FDG) and LN involvement. An integrative review by Usmani et al 19 similarly concluded that FAPI PET/CT outperforms FDG across gynecological malignancies and can potentially influence surgical and systemic treatment strategies. These high-level data situate our small prospective series within a rapidly growing body of evidence that collectively positions FAPI as a promising imaging biomarker in ovarian cancer.

Nevertheless, some limitations should be acknowledged. Our study cohort was small, derived from a single center, and included both staging and restaging patients, introducing heterogeneity that may limit the generalizability of the results. Moreover, although FAPI exhibits low physiologic uptake in the ovaries compared with FDG, false positives have been described in benign fibrotic or inflammatory conditions, such as tuberculosis 20 or fibrous scarring. 8 Ascites without significant stromal cell infiltration may also show low FAPI uptake despite malignant cytology. 21 These pitfalls underscore the need for careful interpretation in the clinical context.

Future directions include prospective multicenter trials to validate diagnostic and prognostic implications, evaluate volumetric and radiomic parameters, and integrate FAPI imaging into surgical planning algorithms. Notably, the theranostic potential of FAPI ligands is increasingly being explored. First-in-human data demonstrated favorable biodistribution and dosimetry of ^177^ Lu-labeled FAP-2286, 23 and early feasibility of FAPI-targeted radionuclide therapy has been reported with encouraging safety and preliminary efficacy. 24 More recently, early clinical experiences, such as the prospective report by Yadav et al 25 on ^177^ Lu-DOTAGA-FAPI dimer therapy in 19 patients with advanced breast cancer, have further underscored the promise of FAPI-based theranostics, demonstrating encouraging efficacy and safety profiles across solid tumors. Although data on ovarian cancer remain lacking, these developments highlight the translational trajectory of FAPI ligands from imaging biomarkers to potential therapeutic agents, which may ultimately extend their clinical impact in gynecological oncology.

Our findings demonstrate that ^68^ Ga-FAPI-04 PET/CT outperforms FDG PET/CT in detecting primary ovarian tumors and peritoneal metastases. Although derived from a small cohort, these results are highly consistent with accumulating prospective evidence and recent meta-analyses. FAPI imaging is promising to improve staging accuracy, guide surgical and therapeutic decision-making, and potentially serve as a theranostic biomarker in ovarian cancer.

## Conclusion

^68^ Ga-FAPI-04 PET/CT demonstrated superior uptake compared with ^18^ F-FDG PET/CT in primary ovarian tumors and PC. Although based on a limited single-center cohort including a mixed staging/restaging population, our findings are consistent with recent prospective studies and meta-analyses supporting the diagnostic advantage of FAPI, particularly for peritoneal disease. These results highlight the potential of FAPI imaging to complement or surpass FDG in staging and follow-up of ovarian cancer, and underscore its promise as a future theranostic tool in gynecologic oncology.
